# Use of Multi-Date and Multi-Spectral UAS Imagery to Classify Dominant Tree Species in the Wet Miombo Woodlands of Zambia

**DOI:** 10.3390/s23042241

**Published:** 2023-02-16

**Authors:** Hastings Shamaoma, Paxie W. Chirwa, Jules C. Zekeng, Abel Ramoelo, Andrew T. Hudak, Ferdinand Handavu, Stephen Syampungani

**Affiliations:** 1 Forest Science Postgraduate Programme, Department of Plant and Soil Sciences, University of Pretoria, Private bag X20, Hatfield, Pretoria 0028, South Africa; 2 Department of Urban and Regional Planning, Copperbelt University, Kitwe 21692, Zambia; 3 Department of Forest Engineering, Advanced Teachers Training School for Technical Education, University of Douala, P.O. Box 1872, Douala, Cameroon; 4 Oliver R Tambo Africa Research Chair Initiative (ORTARChI), Chair of Environment and Development, Department of Environmental and Plant Sciences, Copperbelt University, Kitwe 21692, Zambia; 5 Centre for Environmental Studies (CFES), Department of Geography, Geoinformatics and Meteorology after CFES, University of Pretoria, Private Bag X20, Hatfield, Pretoria 0028, South Africa; 6 USDA Forest Service, Rocky Mountain Research Station, Forestry Sciences Laboratory, 1221 South Main St., Moscow, ID 83843, USA; 7 Department of Geography, Environment and Climate Change, Mukuba University, Kitwe 50100, Zambia

**Keywords:** Miombo woodlands, multi-date, multi-spectral, UAS, object-based, classification

## Abstract

Accurate maps of tree species distributions are necessary for the sustainable management of forests with desired ecological functions. However, image classification methods to produce species distribution maps for supporting sustainable forest management are still lacking in the Miombo woodland ecoregion. This study used multi-date multispectral Unmanned Aerial Systems (UAS) imagery collected at key phenological stages (leaf maturity, transition to senescence, and leaf flushing) to classify five dominant canopy species of the wet Miombo woodlands in the Copperbelt Province of Zambia. Object-based image analysis (OBIA) with a random forest algorithm was used on single date, multi-date, and multi-feature UAS imagery for classifying the dominant canopy tree species of the wet Miombo woodlands. It was found that classification accuracy varies both with dates and features used. For example, the August image yielded the best single date overall accuracy (OA, 80.12%, 0.68 kappa), compared to October (73.25% OA, 0.59 kappa) and May (76.64% OA, 0.63 kappa). The use of a three-date image combination improved the classification accuracy to 84.25% OA and 0.72 kappa. After adding spectral indices to multi-date image combination, the accuracy was further improved to 87.07% and 0.83 kappa. The results highlight the potential of using multispectral UAS imagery and phenology in mapping individual tree species in the Miombo ecoregion. It also provides guidance for future studies using multispectral UAS for sustainable management of Miombo tree species.

## 1. Introduction

The Miombo woodlands are the most extensive dry forest type in southern Africa, with an estimated area of about 2.7 million km^2^ covering Angola, Malawi, Mozambique, Tanzania, Zambia, Zimbabwe, and most of the southern parts of the Democratic Republic of Congo [1]. The woodlands have an estimated 8500 plant species, more than 54 percent of which are endemic. They comprise one of the most important ecosystems in Africa because of their ecological, biological, and socioeconomic significance [2,3,4]. In addition, the Miombo woodlands contribute to the livelihoods of millions of rural and urban dwellers [5]. Some of the local ecosystem’s goods and services include fuelwood, charcoal, timber, fruit, beekeeping, mushrooms, and medicines [3]. These forest ecosystems provide valuable timber resources that support regional economic development, but their ecosystem services have been threatened by climate change and increasing disturbances from deforestation, fragmentation, degradation, and other stressors [2,6]. Trees are the foundational component of the forest ecosystem, and their species composition has important influence on forest biodiversity [7]. Furthermore, tree species composition and spatial distribution are critical information needed to address ecological problems in tropical ecosystems [8]. As a result, accurate information on the spatial distribution of dominant tree species in tropical natural mixed forests with complex distributions and structures, such as the Miombo woodlands, is critical for understanding the dynamics of forest ecosystems. Furthermore, precise mapping of dominant tree species is required for effective management of Miombo woodlands, as well as for characterizing ecosystem services and climate feedbacks on forests [9]. Researchers have mapped tree species composition and distributions to assess biodiversity in other African savanna ecosystems [7,10].

Up-to-date species distribution maps that may be attained from either the application of traditional surveys or remote sensing are critical for sustainable forest resource management [11]. Traditional forest surveys could produce detailed and accurate maps of tree species distributions. However, they are time-consuming, labor-intensive, expensive, and prone to errors that may go undetected [10,12]. Given the difficulties in conducting traditional species mapping surveys [13], remote sensing has emerged as one of the tools for tree species mapping at scales ranging from landscape [14,15] to regional [16,17,18]. The understanding that species have unique spectral signatures associated with characteristic biochemical and biophysical properties can be exploited for mapping plant species using remote sensing [19,20]. Free multispectral imagery, such as Landsat and Sentinel, have low spectral resolutions [21], making them unsuitable for identifying plant species, especially in heterogeneous landscapes, such as the Miombo woodlands, but they can be used for regional species mapping in homogeneous landscapes dominated by planted forests [22]. Hyperspectral imagery, on the other hand, has high spectral resolution with hundreds of contiguous bands across the electromagnetic spectrum, making it more suitable than multispectral imagery for capturing plant biochemical properties, which are closely linked to species identity [19,20], as has been demonstrated in many tree species classification studies across different vegetation formations at landscape scale [10,22,23,24]. However, hyperspectral data are not widely available and remain prohibitively expensive in most Sub-Saharan African countries [25].

To compensate for the low spectral resolution that is common to high resolution imagery (e.g., QuickBird, GeoEye, RapidEye, Pléiades, and WorldView), some studies investigated multi-seasonal imagery for tree species classification [25,26]. One study [25] used two–date WorldView-2 imagery (maximum leaf foliage and transition to senescence) to classify tree species in the South African savannas. Their study compensated for low spectral resolution in WorldView-2 imagery by using two-date WorldView imagery to achieve an overall accuracy (OA) of 80.4% compared to an OA of 76.4% and 72% for maximum leaf foliage and transition to senescence imagery, respectively. Another study [26] investigated the use of multi-season (winter, spring, summer, and autumn) RapidEye imagery for classifying wetland and dryland vegetation communities in Isimangaliso Wetland Park, South Africa’s subtropical coastal region. According to their findings, the four-season imagery combination produced the highest overall classification accuracy (OA = 86 ± 2.8%), followed by the spring (80 ± 2.9%), summer (80 ± 3.1%), autumn (79 ± 3.4%), and winter (66 ± 3.1%). Though the preceding studies demonstrated the ability of high spatial resolution, multi-date imagery to discriminate different tree species in the other African Savanna vegetation formations, none of these studies were conducted within the Miombo ecoregion, which has unique forest structure, species composition, and phenology [27]. Furthermore, very high-resolution spaceborne imagery, such as RapidEye and WorldView, are not flexible enough to capture phenological events that are important for classifying tree species, as cloud cover can be a challenge in the tropics where these species are located. Additionally, the data sets used in these studies are expensive and out of reach for most African savanna researchers and forest managers.

Unmanned Aerial Systems (UAS) have the flexibility of acquiring data almost anytime, anywhere, with limited logistics, making them an essential tool in gathering ultra-high spatial resolution imagery (under 10 cm) on forests for detailed characterization of canopies, which is in contrast to manned airplane and satellite platforms that are less flexible or have fixed acquisition constraints. As a result, using multispectral UAS imagery to classify forest tree species is becoming a popular forestry application [28,29].

The application of UAS imagery for tree species discrimination has shown promising results, as demonstrated in many studies [30,31,32,33,34]. However, all these studies were done in different ecosystems with different tree species, forest structures, and compositions, and therefore, the findings cannot be promulgated to the Miombo ecoregion. Furthermore, [32] observed that the application of UAS imagery for deciduous tree species classification is still at a rudimentary level and recommended that more tests are needed to ascertain its reliability and accuracy. As already stated, species distribution maps are still lacking in the Miombo ecoregion, and remote sensing methods for classifying tree species have not been explored. This study aims to evaluate the potential for multi-spectral and multi-date UAS imagery for classifying the dominant wet Miombo species in Zambia. This study was designed to answer the following research questions:

(i) What is the optimal single season window for acquiring imagery to discriminate tree species in the Miombo ecoregion?
(ii) Could multi-season imagery improve the discrimination of tree species in the Miombo ecoregion?
(iii) What other image features can improve Miombo species classification?

## 2. Materials and Methods

**Figure 1:**
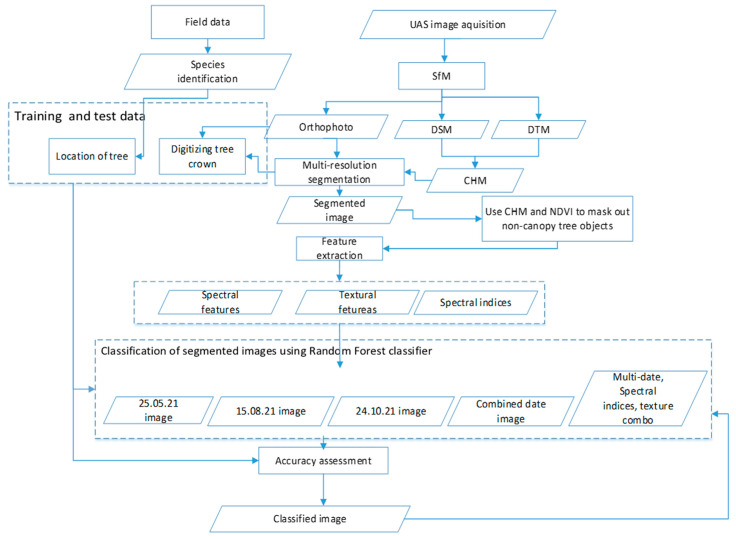
General UAS image acquisition, processing, and classification workflow.

The workflow containing the methodological steps of this study is shown in Figure 1. Within the framework of this study, we acquired multi-date and multi-spectral imagery from multi-rotor UAS combined with individual tree crown delineation algorithms and a machine-learning classifier to identify the dominant tree species in the Miombo woodland of Mwekera area in Zambia.

### 2.1. Study Area

**Figure 2:**
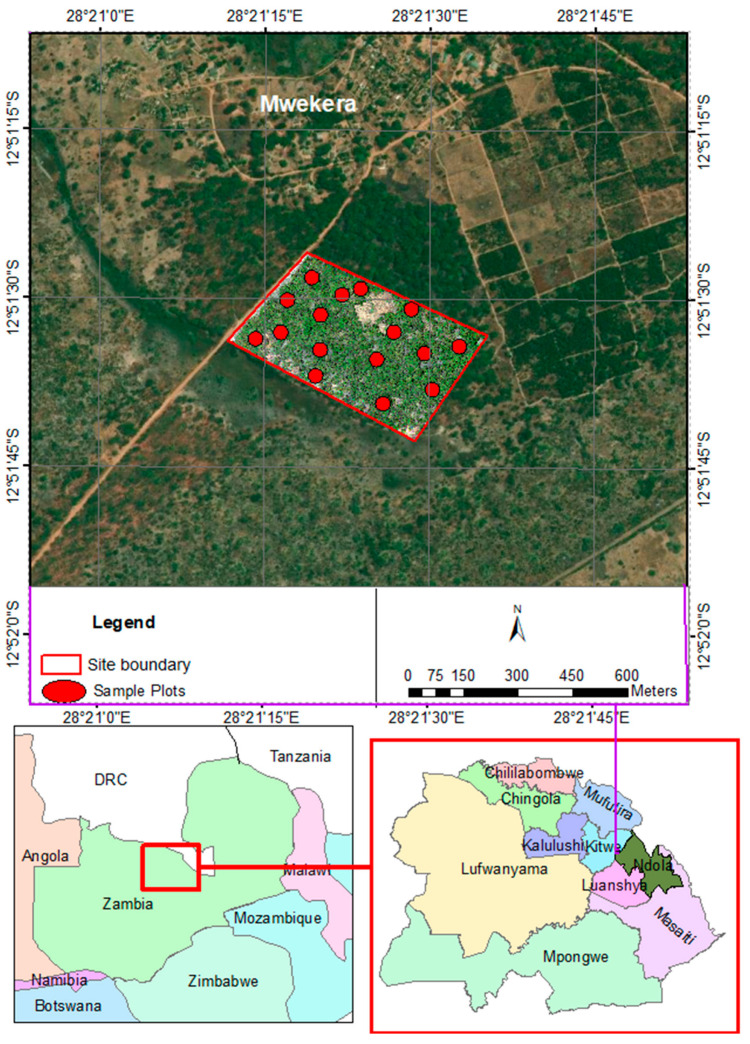
Study area location.

The study area is 22 hectares of wet Miombo woodland located (12.860977 °S, 28.357049 °E; Figure 2) in Mwekera National Forest No. 6, about 15 km southeast of the central business district of the City of Kitwe, in the Copperbelt Province of Zambia. The average human population density in the Copperbelt province is 63.0 persons per km^2^, with an average annual population growth rate of 2.2% (Central Statistical Office, 2012). Mwekera Forest covers about 111 km^2^ and the elevation ranges from 1210 to 1240 m above mean sea level. Annual rainfall ranges between 1000 and 1500 mm and the temperature ranges between 25 °C and 32 °C. The Miombo woodlands, which cover approximately 45% of Zambia, is the predominant vegetation in Mwekera [35]. Mwekera Forest was classified as a National Forest to protect the Mwekera stream catchment, which is part of the Kafue River system.

### 2.2. Field Data Collection

The fieldwork was conducted in May 2021, just before the first flight. Considering the accessibility of the field site and the heterogeneity of tree species, twenty plots of 20 m radius were set up at every 200 m and at additional areas with sudden changes in tree cover in the study area. In each plot, all tree species (Appendix A) with a diameter at breast height (DBH) greater than 5 cm were sampled (N = 688). The attributes of trees collected included individual tree positions, DBH, tree height, and species name. The positions of all the sampled trees were measured using a CHC LT700H real-time kinematic (RTK) Global Navigation satellite system (GNSS) receiver. DBH was measured using a diameter tape and tree height was measured using a Nikon Forest Pro hypsometer. In this study, we conducted our classification experiments based on dominant tree species, which were *Julbernardia paniculata* (JP; 18.5%), *Isoberlinia angolensis* (IA; 16.6%), *Marquesia macroura* (MM; 15.7%), *Brachystegia longifolia* (BL; 9.3%) and *Brachystegia spiciformis* (BS; 7.4%) (Table 1; Appendix A). The remaining species were recorded in less than 5% of the samples and were, therefore, not considered for classification. Furthermore, the dominant species found in Mwekera (Table 1), except for *Marquesia macroura*, were found to be preferred charcoal species [36], which makes the site vulnerable to over-exploitation.

### 2.3. UAS Image Data Acquisition

Three UAS images used to classify tree species were acquired on 25 May 2021 at full leaf maturity, 15 August 2021 at senescence for the majority of dominant canopy tree species and early flushing for *BL* and *BS* species, and 24 October 2021 at greening of flushed leaves for the majority of dominant species [1,37]. The DJI Phantom 4 RTK Multispectral multi-rotor UAS, equipped with one RGB camera and a multispectral camera array with five cameras covering blue (450 nm ± 16 nm), green (560 nm ± 16 nm), red (730 nm ± 16 nm), red edge (450 nm ± 16 nm), and near-infrared (840 nm ± 26 nm), as well as a D-RTK 2 mobile Global Navigation Satellite System (GNSS) base station [38], was used to capture imagery for this study. This UAS was chosen for our study because of two capabilities: (i) Real Time Kinematic GNSS capability that enabled direct image georeferencing for easy processing and comparison of multi-date images, and (ii) integrated sunlight sensor for consistency of images collected at different times of the day. All our flights were undertaken between 11:30 a.m. and 12:30 p.m. local time to minimize shadowing on the images. In order to ensure consistent comparisons between the multi-date UAS imagery, the same UAS flight parameters were applied on all dates (Table 2).

### 2.4. UAS Data Pre-Processing

The UAS images from the three dates were processed using the Structure from Motion (SfM) approach [39] based on the workflow in Agisoft Metashape software version 1.7 [40], and can be summarized as follows: (i) photos were uploaded while selecting the multi-camera system, and bands were arranged according to image metadata, (ii) the reflectance was calibrated based on the sun sensor, (iii) photos were by estimating the camera position of the multi-camera system, and sparse point clouds were generated consisting of tie points and the estimated interior orientation parameters for each sensor, (iv) a dense point cloud was generated based on the calculated exterior and interior orientation parameters using dense stereo matching to densify the point clouds, (v) a Digital Surface Model (DSM) was generated based on the dense point cloud and resolution, (vi) an orthophoto mosaic was generated based on the DSM, and (vii) the orthophoto mosaic was exported in Geotiff format. The other process performed with Metashape software was to classify ground points and generate a digital terrain model (DTM), which was also exported together with the DSM for further processing in the calculation of the canopy height model (CHM). In order to optimize on storage space and processing time, the orthophoto mosaic, DSM, and DTM were exported at a degraded resolution of 0.30 m, which was tried and found suitable for segmenting tree crowns of mature deciduous trees [41].

### 2.5. Computation of the CHM

The CHM was computed based on recommendations from [42], who found combination of UASs with non-radiometric RGB sensors and the SfM approach (UAS-SfM) to generate better DTMs in open woodlands compared to closed woodlands, due to the inability of optical UAS imagery to capture the ground in closed canopy woodlands. Similar observations were made by [43], who used leaf-off UAS-SfM derived DTMs as ground reference for supporting teak plantations’ inventory in the dry forests of the coastal region of Ecuador. A study by [44] assessed tree damage in a West Virginia Research Forest using leaf-on generated UAS-SfM DSM and leaf-off DTM. Therefore, we took advantage of our multi-date data set to generate the best possible CHM from our available data sets by subtracting the leaf-off (15.08.21) DTM from leaf-on (25.0522) DSM. The computed CHM was resampled to 0.3 m resolution to match the orthophoto and used an input in the tree species classification process.

### 2.6. Tree Species Classification

The tree species were classified using object-based image analysis (OBIA) [45,46]. This method outperforms pixel-based methods for classifying tree species from high-resolution imagery [47]. Therefore, OBIA was used in this study, and it was performed in three steps: image segmentation, feature extraction, and image classification.

#### 2.6.1. Image Segmentation

The orthophoto images were processed into homogeneous segments that closely correspond with individual tree crowns using the multi-resolution algorithm [48] implemented in eCognition Developer version 9.0 (Trimble) software. This algorithm grows by merging one pixel with neighboring pixels based on spectral and/or shape similarity criteria. A combination of orthophoto and CHM was assessed in this study as CHM was found to improve individual tree segmentation in other studies [49,50]. The UAS imagery captured in May (leaf-maturity) was used for segmentation since all Miombo trees have a well-defined tree crown shapes at this stage of the year. Multiple iterations were performed via trial and error by varying the shape, compactness, and scale parameters, and comparing to the resulting tree crowns. Furthermore, the effect of combining the orthophoto and CHM to the segmentation result was also assessed. The result of the segmentation were polygons of homogeneous objects representing a tree crown or group of similar tree crowns. The image objects polygons generated were used as a basis for segmenting the August UAS orthophoto (senescence for most of the Miombo tree species) and October UAS orthophoto (leaf-flushing for Miombo tree species). This was done to make sure that we used the same tree objects when comparing the accuracy of the classification results from the three image dates.

##### Segmentation Accuracy Assessment

The accuracy of OBIA analysis is based on the accuracy of the segmentation process and it is therefore important to assess the quality of the segmentation before proceeding to the subsequent processes of feature extraction and image segmentation. In this study, the area estimation technique described in [51] was used to assess the segmentation accuracy of tree crowns. The three measures were compared to assess the accuracy of the tree crown segmentation using the following equations

(1) $$\mathrm{Oversegmentation}\;\left(\mathrm{OS}\right)=\frac{area\left(ARP\cap^{\;}ADP\right)}{area\left(ARP\right)}$$

(2) $$\mathrm{Undersegmentation}\;\left(\mathrm{US}\right)=\frac{area\left(ARP\cap^{\;}ADP\right)}{\left(ADP\right)}$$

(3) $$\mathrm{Segmentation}\;\mathrm{error}\;\left(\mathrm{SE}\right)=\surd \left(\frac{{\left(OS\right)}^{2}+{\left(US\right)}^{2}}{2}\right)$$

where *ARP* is a detected object area segmented by the MRS algorithm that is one-to-one with a reference polygon; *ADP* is the area of the reference polygon (tree crown), which is manually digitized in ArcMap (ArcGIS Desktop Version 10.7.1); [52] and area (*ARP* ∩ *ADP*) is the area of the manually delineated polygons correctly identified by the MRS algorithm. The ideal value of the oversegmentation, undersegmentation, and total detection error is 0. The reference polygons (tree crowns) were manually digitized in ArcGIS for two forest stands and then applied to quantify the segmentation error.

#### 2.6.2. Feature Extraction

Before classification of tree species, it is essential to extract features of segmented tree objects that are used to discriminate different tree species in the subsequent classification process [50]. The first step in our feature extraction process was to mask off non-canopy tree objects from canopy tree objects so that only features related to canopy tree objects are considered for subsequent tree species classification. This was done by applying a threshold height of greater than 3 m of CHM to represent canopy tree objects. 

The non-canopy tree objects taller than 3 m were separated by using normalized difference vegetation index (NDVI) value of less than 0.1. We explored the use of a combination of spectral, texture, and vegetation indices because use of multiple features have been found to improve tree species discrimination in other studies [15,33,53]. All the canopy tree object features for all the three dates were extracted in eCognition Developer software before exporting to ArcGIS for tree species classification. The extracted features built into eCognition Developer software [54] included: spectral features (mean blue, mean green, mean red, mean red-edge, mean near infra-red (NIR), grey level co-occurrence matrix (GLCM) textural features (contrast, correlation, dissimilarity, and standard deviation), and band metrics (mean brightness and maximum difference). The vegetation indices included: green chromatic coordinate (GCC), red chromatic coordinate (RCC), and NDVI, which were computed and extracted within eCognition software using the equations in Table 3.

The tree objects were exported from eCognition as shape files with all the extracted features as attributes. The shape file attributes of the exported object features were rescaled by normalizing them to a common scale in order to prevent attributes with high range values from dominating those with low range values during the classification process [57]. All feature values were rescaled to a range of 0 to 1 in ArcMap using the attribute table field calculator (Equation (1)). The shape files were converted to raster in ArcMap with each feature been used to create a single band raster image.

(4) $$rescaled\;value=\frac{\left(feature\;value-minmum\;value\right)}{(maximum\;value-minmum\;value}$$

#### 2.6.3. Species classification

The tree species classification was done using Random Forest (RF), a non-parametric machine learning classifier that has been used widely in tree species classification using very high resolution imagery [26,31,32,50,58]. RF uses training samples, validation samples, and the majority vote to classify an object into a specific class. In the current study, the RF was implemented in ArcMap. The training and validation sample image objects were collected using the training sample manager in ArcMap guided by field sample crowns, but only sunlit objects were collected to represent a pure sample for each tree species, and a shadow class was added to classify shadowed areas. A total of 344 training samples were collected for the six classes divided as follows: JP (89), IA (80), MM (76), BL (45), BS (45), and shadow (19). The sample data were randomly split into training (70%) and validation (30%). The same training and validation samples were used to train and validate classification results for single-date imagery, multi-date and multi-feature image combination to find the optimal solution for discriminating different tree species within the Miombo woodland study area.

##### Class Separability

The separability of the 6 classes was summarized by collecting mean statistics of training data for each class in ArcMap Training Sample Manager and exporting to Excel for plotting and visualization. The variability of spectral, vegetation indices, and texture features across dates and image combinations were visualized to assess the separability of different species.

##### Classification Accuracy Assessment

The effectiveness of the different image date combinations to discriminate different tree species was assessed using a confusion matrix. For each classification result, the producer’s accuracy, user’s accuracy, overall accuracy, and kappa statistics were computed to assess the ability to discriminate species.

## 3. Results

### 3.1. Identifying Segmentation Parameters

**Figure 3:**
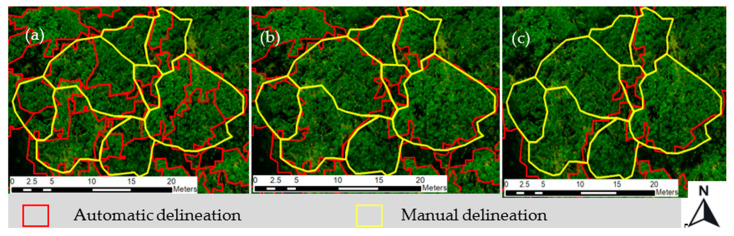
Visual comparison of segmentation using different scale parameter: (**a**) 50 (oversegmentation); (**b**) 80 (correct segmentation); and (**c**) 150 (undersegmentation).

**Figure 4:**
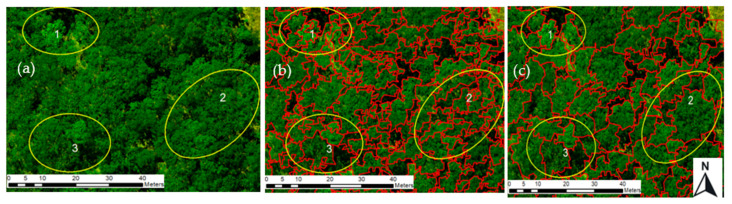
Visual comparison of segmentation using orthophoto alone vs. orthophoto with CHM at highlighted sites 1–3: (**a**) Original orthophoto; (**b**) using only the orthophoto, over-segmentation with irregular outlines for tree crowns; and (**c**) using orthophoto and CHM, tree crowns are well segmented with smoother outlines.

In this study, after a systematic trial and error process, the suitable segmentation parameter combinations for delineating tree crowns were scale (90), shape (0.8), and compactness (0.9). Scale was found to be the most sensitive parameter, and the effect of changing the scale while keeping the other parameters the same was evaluated by visual comparison. This showed that when the scale factor was 50, tree crowns were over-segmented; when the scale factor was 150, tree crowns were under-segmented; and when the scale factor was 80, tree crowns were best segmented (Figure 3). We also compared the CHM’s contribution to segmentation visually in Figure 4 and quantitatively in Table 4.

### 3.2. Discrimination of Dominant Tree Species

The investigated image features (mean spectral bands, mean spectral indices, and GLCM textural features) used for discriminating tree species revealed that spectral indices performed better than other image features ( Appendix B). The performance of each image feature in discriminating the tree species for each of the image dates is indicated below.

**Figure 5:**
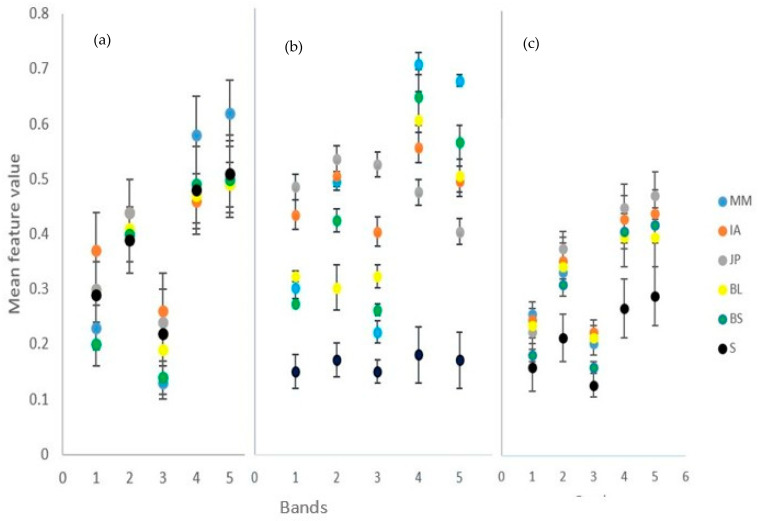
Species separability in different bands (1, blue; 2, green; 3, red; 4, red-edge; 5, near infrared): (**a**) 25.05.21 image, (**b**) 15.08.21 image; and (**c**) 24.10.21 image. S = shadow.

Figure 5 shows the variability in the mean spectra across the three image dates. Figure 5a (May image): in the blue band, JP, BL, and shadow were mixed, while IA, BS, and MM were discriminable; in the green band, only JP stood out with relatively high reflectance and all the other species were mixed with shadow; in the red band, BL was discriminable, JP, IA, and shadow were somewhat mixed, while BS and MM were mixed; and in the red-edge and NIR bands, only MM was discriminable, with all other species mixed with shadow. In Figure 5b (August image): the shadow was discriminable from all the species across the five bands; all the dominant species were clearly discriminable in the red and red-edge bands; in the blue band, JP and IA were discriminable, while BS, BL, and MM were somewhat mixed; in the green band, BS and BL were discriminable, while MM, IA, and MM were somewhat mixed; and in the NIR band, JP, MM, and BS were discriminable, while IA and BL were somewhat mixed. In Figure 5c (October image): the shadow was discriminable from all the tree species in all the bands except for in the blue band, where it was somewhat mixed with BS; in the blue band, BL, JP, AI, and MM were mixed; in the green band, all the species were mixed; in the red band, only MM was discriminable with the rest of the species somewhat mixed; and in the red-edge and NIR bands, MM, BL, and BS were mixed, while IA and JP were discriminable (description summarized in Appendix B).

**Figure 6:**
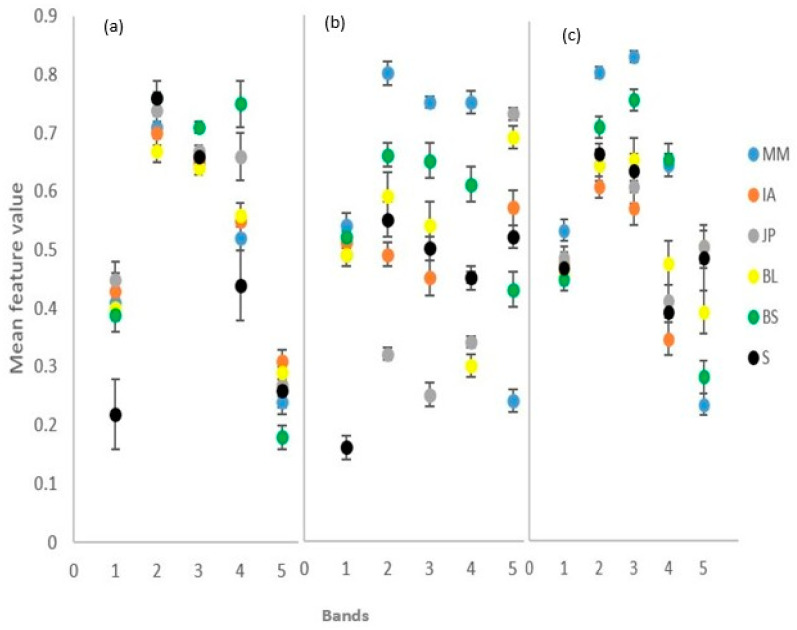
Species separability in band spectral metrics bands (1, brightness; 2, maximum difference; 3, NDVI; 4, GCC; 5 RCC): (**a**) 25.05.21 image, (**b**) 15.08.21 image; and (**c**) 24.10.21 image. S = shadow.

Figure 6 shows the variability in the extracted spectral indices features across the three image dates, which revealed improved species separability compared to raw spectral band data. Figure 6a (May image): in the brightness band, only the shadow was discriminable, with all the species mixed due to uniform brightness in all species at leaf maturity; maximum difference band, all the species were mixed with shadow; BS was discriminable in the NDVI band, while all other species were mixed with shadow; in the GCC band, shadow, JP, and BS were discriminable, while MM, IA, and BL were mixed; and in the RCC band, only BS was discriminable, while the rest of the species were mixed with shadow. In Figure 6b (August image): the shadow was discriminable from all the species across all spectral metrics bands except in GCC, where it was mixed with IA; all the dominant tree species were discriminable in the NDVI, RCC, and maximum difference bands; and in the GCC band, all species were discriminable except IA, which was mixed with shadow. In Figure 6c (October image): only MM was discriminable in the brightness band, with the rest of the species somewhat mixed with shadow; in the maximum difference band, IA, BS, and MM were discriminable, while JP and BL were somewhat mixed with shadow; in the NDVI band, JP, IA, BS, and MM were discriminable, while BL was somewhat mixed with shadow; in the GCC band, BL and IA were discriminable, while JP was mixed with shadow, and BS was mixed with MM; and in the RCC band, BL, BS, and MM were discriminable, while shadow, JP, and IA were mixed (description summarized in Appendix C).

**Figure 7:**
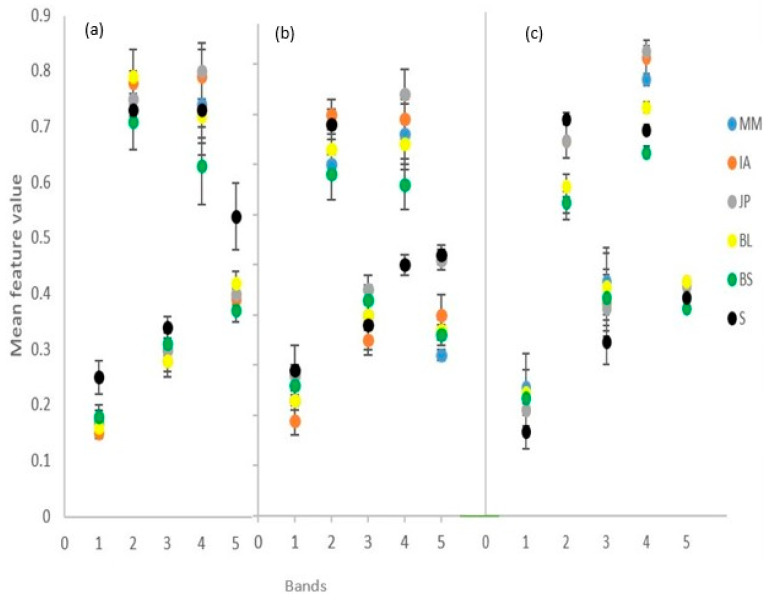
Species separability in GLCM texture bands (1, contrast; 2, correlation; 3, dissimilarity; 4, entropy; and 5, standard deviation): (**a**) 25.05.22 image, (**b**) 15.08.21 image; and (**c**) 24.10.21 image. S = shadow.

Figure 7 shows the variability in the extracted GLCM texture features across the three image dates, which exhibited more mixing among species compared to other considered features. Figure 7a (May image): the shadow is discriminable in the contrast and standard deviation bands, BS was discriminable in the entropy band, and the rest of the species were mixed in the rest of the bands: In Figure 7b (August image): the shadow and JP were discriminable in the entropy band, while in the rest of the bands the classes were mixed. In Figure 7c (October image): shadow was discriminable in all bands except the standard deviation band; BS, BL and MM were discriminable in the entropy band; JP was discriminable in the correlation band; while in the rest of the bands, the classes were mixed (description summarized in Appendix D).

### 3.3. Tree Species Classification

**Figure 8:**
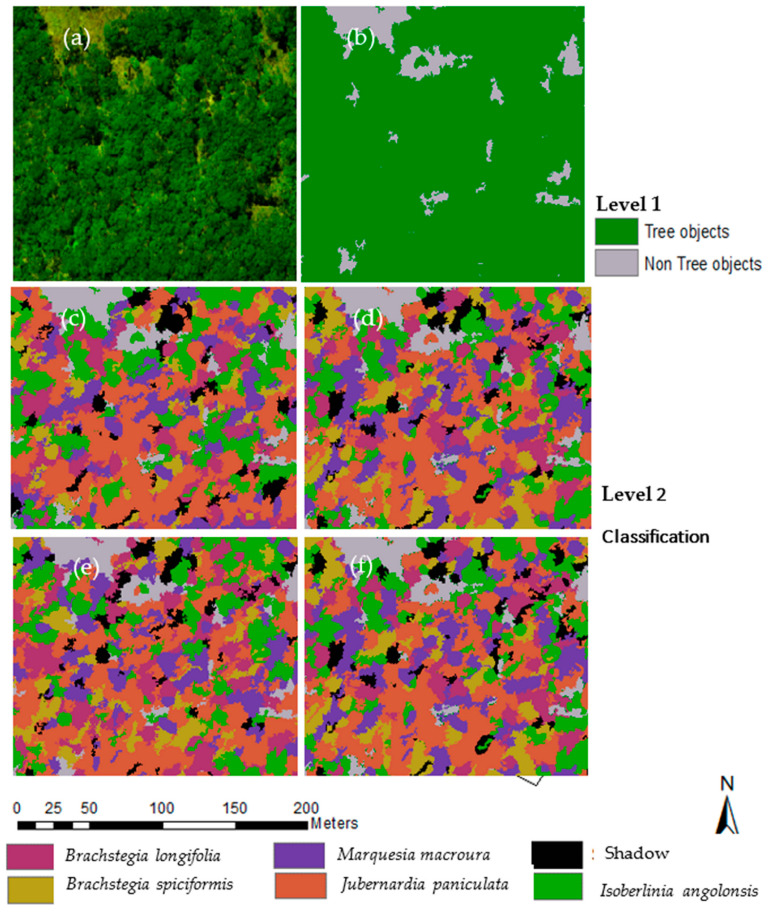
Classification of dominant tree species: (**a**) orthophoto mosaic at leaf maturity; (**b**) level 1 classification to separate trees from non-tree objects; (**c**) species classification at leaf maturity (May image); (**d**) species classification at transition to senescence (August image); (**e**) species classification at flushing of new leaves; and (**f**) species classification using multi-date and multi-feature image combination.

Figure 8 presents the results of the tree species classification using the Random Forest algorithm. The visual observation indicated that JP occupied the most significant distribution across the entire study area. Figure 8b depicts the results of canopy species and herbaceous layer discrimination using data fusion of UAS CHM and multi-spectral orthophoto mosaic, while Figure 8c–e show the classification results from the May, August, and October images, respectively. Figure 8f shows the classification results of the best combination of multi-date and multi-feature images considered in the study.

The confusion matrix of the five dominant tree species using the three groups of metrics is shown in Table 5. In general, using single date data, the accuracy of the tree species classification, except for *Marquesia macroura*, is higher in the August image (overall accuracy: 80.12 %, kappa accuracy: 68%), followed by the May image, with the October image being the least accurate. In addition, the average producer’s accuracy (PA) and user’s accuracy (UA) for all the dominant species were above 75%, which points to good spectral discrimination among species in the August image when JP is in senescence, while BS and BL are flushing and have a distinctive reddish color. Furthermore, the species were poorly separable in the October image, with BS, BL, and MM mixing across all bands and yielding an average PA and UA of less than 60%. Using multi-date images improved the tree species classification accuracy by about 4% to 84.25% OA and 0.72 kappa. Additionally, combining multi-date images, spectral indices, and texture improved the classification accuracy to 87.07% OA and 0.83 kappa.

## 4. Discussion

### 4.1. Segmentation of Tree Crowns

The segmentation of tree crowns in this study was completed using the MRS algorithm iteratively via trial and error method by varying the scale, shape, and compactness parameters. The suitable parameters for delineating tree crowns in this study were 90, 0.8, and 0.9 for scale, shape, and compactness, respectively. Among these parameters, the scale parameter was found to be the most sensitive and it substantially affected the segmentation results. This observation is consistent with the findings of studies by [41] in a mixed forest in Amstelveen, Germany, and [50] in a mixed forest in Xugongqing, Dêqên,, Yunnan province China. The combination of multi-spectral orthophoto and CHM improved the segmentation accuracy by 6% compared with using only the multi-spectral orthophoto (Table 4). This improvement in segmentation accuracy can be attributed to the addition of the three-dimensional structural information of the trees and the CHM. Such observations have also seen in Arizona, United States of America (USA) [59] and Qi’ao Island, China [15], both of which demonstrated the importance of tree height to improve the segmentation accuracy in natural forest stands.

The tree crown segmentation accuracy obtained in this study is within the range (60% to 95%) reported in other deciduous forests [41,50] However, the accuracy of the tree crown segmentation may be dependent on many factors, including image acquisition date and stand structure in different sites For example, [60] applied a local maxima method to UAS-derived CHM to delineate individual tree crowns across a boreal forest, achieving accuracies between 40% and 95%, depending on the characteristics of the site. Another study by [61] used a combination of spectral and point cloud UAS data through sub-crown k-means clustering where 48% of the individual tree crown were correctly detected and segmented across a complex forest ecosystem. They also experimented using the same technique with CHM only and observed an accuracy degradation of 4.1%, thus confirming the observation elsewhere [50] that the synergy between CHM and spectral information gives superior results compared to a single data set approach.

### 4.2. Optimal Single Date Imagery

The August image (Figure 5b) was identified as the best single date image for discriminating tree species in the wet Miombo woodlands. August–September coincides with the transition to senescence for most of the dominant wet Miombo tree species and early flushing for some species in the *Brachystegia* genus [1]. Moreover, interspecies phenological differences are more pronounced during this period, which maximizes interspecies spectral variability, a key feature for separating tree species [62]. JP was strongly separable across all spectral bands in the August image, resulting in high producer’s and user’s accuracies compared to other species, and exhibited characteristics of a species in senescence, with high reflectance in the visible part of the spectrum and low reflectance in the red-edge and NIR part of the spectrum. In contrast, MM and BS exhibited the characteristics of species at leaf flushing, with low reflectance in the pigment absorption bands (blue and red) and high reflectance in the red-edge and NIR bands. These results are consistent with findings in the study by [25], who also reported better classification accuracy in the image acquired during transition periods from full green canopy to senescence in the South African savannah. These findings corroborate earlier works in other regions by [63] in West Virginia, USA, [62] in Monks Wood, Cambridgeshire, eastern England, and [64] in Hawai’i Volcanoes National Park, Hawai’i, USA. The October image, which coincided with the period when newly flushed leaves turn green in the wet Miombo woodlands [1,65], resulted in the lowest accuracy (Table 5) due to low interspecies spectral variability at this phenological stage. These results contrast with the findings by [31], who found early summer to be the optimal single date imagery for discriminating deciduous tree species in Grand-Leez municipality, Belgium. The differences in findings could be attributed to differences in species composition in the two regions.

### 4.3. Improved Accuracy with Multi-Date Image

The high accuracy achieved in the multi-date image compared to single date images (Figure 6 and Table 5) suggests that multi-date imagery takes advantage of interspecies differences in phenologies, exhibiting different spectral characteristics for tree species on different dates, which compensate for the low spectral resolution [63] of the UAS imagery used in this study. Furthermore, it demonstrates that using a single date image results in missing important information that can be used for tree species discrimination. The improvement in the classification results using multi-date imagery is in agreement with the observations in other studies elsewhere [25,26,62], who found that utilizing multi-date image data improves the spectral variability among species because of the differences in the phenological developments of different species across the seasons. Additionally, [31] captured multispectral UAS imagery at strategic dates of phenological development for 130 hectares of broadleaved forest in Grand-Leez, Belgium. They used the Random Forest (RF) classification approach to classify five deciduous species groups using single-date, two-date, and three-date multispectral image combinations and observed that the three-date combination yielded superior results compared to the others.

### 4.4. Image Indices Improve Classification Accuracy

The addition of spectral indices increases separability of different classes as opposed to just using raw spectral information. For example, the BS and shadow classes, which were difficult to separate using raw spectral information (Figure 5) in the May image, become very separable using the spectral indices (Figure 6), thus demonstrating that a combination of raw spectral bands and spectral indices, even for a single date image, has potential to improve classification accuracy. These findings corroborate works by [50] in China and [66] in Brazil on how spectral indices improve the classification accuracy of tree species. This highlights the importance of using a combination of raw spectral data and derived features, such as texture and spectral indices, when classifying tree species, especially when using images of lower spectral resolution. This was in contrast to the findings of [26], who reported no improvements in vegetation community classification when spectral indices were used. Our study shows that the mixing of species when texture features are used for tree species classification (Figure 6) results in low classification accuracies. This is in line with a study by [67], who found that when combined with spectral features, GLCM textural features did not improve the classification accuracy of tree species in two observed sites in China (homogeneous park forest and heterogeneous management forest). However, our study contradicts studies by [33,68,69,70], who observed that texture features improve tree species discrimination. The differences in results could be attributed to the similar appearance of the Miombo woodlands species [1], which translates to a similar texture.

The methods proposed add a new technique for mapping of Miombo woodland tree species targeted for various products at a local scale. For instance, all the dominant Miombo species identified in this study are targeted for fuelwood production because of their burning qualities [5], *Isoberlinia angolensis* is targeted for timber, and *Brachystegia longifolia* is targeted for its bark rope; these qualify them as candidates for conservation and sustainable utilization [36]. The classification results attained using multi-date UAS imagery for the dominant Miombo species unlocks the potential for mapping and monitoring their distribution, as well as to inform decision making for better management and conservation. Although the study was limited to a small site and a few species, site-specific studies confined to one or a small group of species are important for upgrading existing information, and thus help sustainable use and management of forest resources [2]. Therefore, the approach used here can be a turnkey for species distribution mapping in the Miombo, as well as other regions and ecosystems, to supplement already existing methods that are used in the conservation of tree species that are important for the desirable goods and ecosystem services that they provide.

## 5. Conclusions

This study investigated the potential for using multi-spectral UAS imagery in classifying the dominant tree species of the wet Miombo woodlands. Single dates, combination of dates, and combination of features were used in the classification of tree species, as all of these tend to influence the classification accuracy. The August image achieved the best single date accuracy (80.12% OA, 0.68 kappa), compared to the October (73.25% OA, 0.59 kappa) and May (76.64% OA, 0.63 kappa). The use of a multi-date image combination improved the classification accuracy to 84.25% OA and 0.72 kappa. After the addition of spectral indices, the accuracy was further improved to 87.07% and 0.83 kappa. The use of multi-date imagery was found to be very useful in capturing the interspecies phenological differences that are useful for identifying different tree species in the Miombo woodlands. The study has demonstrated the applicability of multi-spectral UAS imagery and OBIA to classify tree species in the Miombo woodlands

The results have implications on the choice of dates for image acquisition for natural resources managers using multi-spectral UAS imagery to map tree species in the Miombo woodlands and elsewhere. Judging by the variation in species separability across different dates, it seems imperative to acquire imagery on seasonally separated dates that will enable the capture of all of the important phenological traits that are important for separating tree species using spectral information. Specifically, denser image acquisition dates should be concentrated around July–September for the Miombo woodland, as this is when most of the dominant tree species here are in transition from mature leaves through senescence to flushing. Due to the phenological variation of the Miombo woodland tree species, no single date imagery can outperform the broadly spread multi-date imagery combination in capturing the information required for separating different tree species.

## Figures and Tables

**Table 1:** Sampled dominant tree species in the area.

Species Code	Tree Species	Common Local Uses	Trees Sampled	Training Samples	Validation Samples
JP	*Julbernardia paniculata*	Charcoal, pole, timber	127	89	38
IA	*Isoberlinia angolensis*	Charcoal, timber, pole	114	80	34
MM	*Marquesia macroura*	Poles, charcoal	108	76	32
BL	*Brachystegia longifolia*	Charcoal, bark rope	64	45	19
BS	*Brachystegia spiciformis*	Charcoal, bark rope	51	36	15

**Table 2:** Imagery acquisition parameters.

UAS Flight Parameters	Value
Camera model	DJI P4 Multi-spectral
Flight height (m)	100
Flight speed (m/s)	5
Forward overlap (%)	85
Side Overlap (%)	75
Ground resolution (m)	0.05
Spectral bands	Blue, green, red, red-edge, near infrared
Time of flight	11:30 a.m.–12:30 p.m.

**Table 3:** Equations of vegetation indices used.

Vegetation Index	Equation	Source
NDVI	NDVI = (nir − red)/(nir + red)	[55]
GCC	GCC = green/(blue + green + red)	[56]
RCC	(RCC = red/(blue + green + red)	[56]

**Table 4:** Segmentation accuracy of using UAS orthophoto and combination of UAS orthophoto and CHM.

Image Source	OS	US	SE	Accuracy (%)
Orthophoto	0.26	0.17	0.22	78
Orthophoto and CHM	0.17	0.14	0.16	84

OS = oversegmentation, US = undersegmentation, SE = segmentation error.

**Table 5:** Comparison of classification accuracies of tree species for single date, multi-date, and multi-feature imagery.

Classes	25.05.21 Spectral		15.08.21 Spectral		24.10.21 Spectral		Multi-Date Spectral		Multi-Date SELECTION (Spectral and Indices)	
	PA%	UA%	PA%	UA%	PA%	UA%	PA%	UA%	PA%	UA%
JP	61.42	53.56	93.21	84.74	79.61	72.00	95.11	93.17	96.50	96.03
IA	73.34	80.05	77.23	80.41	65.20	76.24	84.05	92.50	87.17	85.22
MM	82.44	88.25	70.08	67.45	54.17	60.58	93.86	84.35	94.88	86.24
BL	58.22	67.45	86.08	79.44	57.28	44.56	86.75	72.04	92.15	85.36
BS	74.31	71.25	75.41	81.98	52.5	65.05	91.15	82.15	95.04	81.26
S	65.62	67.15	98.20	100	88.75	86.30	90.52	96.01	97.42	100
OA%	74.64		80.12		68.25		84.25		87.07	
Kappa	0.63		0.68		0.59		0.72		0.83	

Abbreviations: JP = *Julbernardia paniculata*, IA = *Isoberlinia angolensis*, MM = *Marquesia macroura*, BL = *Brachystegia longifolia*, BS = *Brachystegia spiciformis*, S = shadow.

## Data Availability

The data are available on request from the corresponding author.

## Appendix A. Sampled Tree Species in the Study Area

**Tree Species**	**N**	**%**	**DBH (cm)**		**TH(m)**	
			**Mean**	**Range**	**Mean**	**Range**
*Julbernardia paniculata*	127	18.5	31.03	13.5–59.90	17.79	8.50–25.00
*Isoberlinia angolensis*	114	16.6	23.92	9.90–44.70	14.55	5.00–20.50
*Marquesia macroura*	108	15.7	29.21	5.30–70.00	15.10	3.25–25.00
*Brachystegia longifolia*	64	9.3	20.65	11.8–64.00	11.27	8.50–23.00
*Brachystegia spiciformis*	51	7.4	18.55	5.00–64.20	9.97	5.80–20.50
*Parinari curatellifolia*	18	2.6	23.48	6.00–53.50	13.67	6.00–24.00
*Ochna pulchra*	17	2.5	7.62	5.20–10.90	5.70	4.50–8.00
*Baphia bequaertii*	16	2.3	11.63	5.80–23.70	6.95	3.00–15.00
*Pericopsis angolensis*	16	2.3	24.42	10.3–70.00	14.01	5.00–25.10
*Diplorhynchus condylocarpon*	14	2.0	8.94	5.00–18.00	7.64	4.50–10.00
*Anisophyllea boehmii*	11	1.6	18.77	5.10–44.90	11.74	3.75–19.50
*Erythrina abyssinica*	11	1.6	18.05	8.60–33.30	10.21	5.30–20.50
*Hymenocardia ulmoides*	8	1.2	24.05	5.40–9.90	19.94	4.50–7.00
*Pseudolachnostylis maprouneifolia*	7	1.0	22.04	7.00–20.80	11.64	5.00–10.00
*Syzygium cordatum*	7	1.0	21.20	9.10–19.20	11.21	5.25–10.00
*Hexalobus monopetalus*	7	1.0	14.13	5.80–57.30	7.94	4.75–22.00
*Pterocarpus angolensis*	7	1.0	12.29	5.30–28.10	8.22	5.30–15.00
*Swartzia madagascariensis*	7	1.0	8.16	5.50–10.80	5.34	3.30–8.75
*Diospyros batocana*	4	0.6	10.75	9.00–11.60	10.13	7.00–17.50
*Burkia africana*	4	0.6	8.55	7.80–9.30	6.38	6.25–6.50
*Albizia adianthifolia*	4	0.6	14.15	12.3–18.00	13.00	10.75–16.50
*Uapaca sansibarica*	4	0.6	15.23	8.90–22.00	10.44	6.00–15.75
*Lannea discolor*	4	0.6	13.73	5.50–23.50	9.58	5.00–14.50
*Diospyros mespiliformis*	4	0.6	19.73	19.1–20.90	12.30	12.30–12.30
*Brachystegia floribunda*	4	0.6	34.45	25.7–44.50	19.00	17.50–20.00
*Mapraunea africana*	3	0.4	8.90	6.80–10.60	5.83	4.25–6.75
*Bobgunnia madagascariensis*	3	0.4	7.90	7.50–8.70	4.50	4.25–5.00
*Dalbergia nitidula*	3	0.4	27.93	22.0–30.90	13.17	13.00–13.25
*Strychnos innocua*	3	0.4	7.27	6.40–7.70	6.78	5.35–7.50
*Pseudochnostylis maprouneifolia*	3	0.4	7.77	5.80–11.60	5.87	5.30–7.00
*Maprounea africana*	3	0.4	8.90	6.80–10.60	5.83	4.25–6.75
*Rhus longipes*	3	0.4	9.43	8.80–9.90	5.50	5.00–6.00
*Albizya adiansfolia*	3	0.4	18.43	7.80–26.70	13.08	6.75–17.50
*Combretum zeyheri*	2	0.3	23.65	17.7–29.60	12.00	9.00–15.00
*Faurea speciosa*	2	0.3	8.90	8.90–8.90	5.75	5.75–5.75
*Magnistipula butayei*	2	0.3	15.90	15.9–15.90	8.00	8.00–8.00
*Erythropeleum africanum*	2	0.3	30.10	30.1–30.10	17.75	17.75–17.75
*Ochna schweinfurthiana*	2	0.3	6.80	6.60–7.00	5.95	5.00–6.90
*Albizia antunesiana*	2	0.3	32.40	21.6–43.20	17.90	17.50–18.30
*Albizia versicolor*	2	0.3	33.50	33.5–33.50	11.25	11.25–11.25
*Phyllocosmos lemaireanus*	2	0.3	5.75	5.70–5.80	6.13	5.75–6.50
*Uapaca kirkiana*	2	0.3	14.35	8.90–19.80	9.50	5.50–13.50
*Harungana madagascariensis*	1	0.1	5.70	5.70–5.70	4.50	4.50–4.50
*Canthium crassum*	1	0.1	37.00	37.0–37.00	22.00	22.00–22.00
*Oxtenanthera abyssinica*	1	0.1	9.20	9.20–9.20	11.00	11.00–11.00
*Dallbegiella nyasae*	1	0.1	33.30	33.3–33.30	17.25	17.25–17.25
*Monotes africanus*	1	0.1	7.20	7.20–7.20	10.75	10.75–10.75
*Syzygium guineense*	1	0.1	5.90	5.90–5.90	6.70	6.70–6.70
*Uapaca nitida*	1	0.1	14.60	14.6–14.60	6.00	6.00–6.00
*Albizya atunizyana*	1	0.1	7.50	7.50–7.50	7.75	7.75–7.75
Total	688	100				

## Appendix B. Summary of Class Separability Using Mean Spectral Features across the 3 Sampled Dates

**Bands**	**Separable Classes**	**Mixed Classes**	**Date**
Blue	IA, BS, MM	BL, JP and shadow	25.05.21
Green	JP	IA, BS, BL, MM, Shadow	
Red	BL	JP, IA and shadow/ BS, MM	
Red-edge	MM	IA, BS, BL, JP, shadow	
Near infrared	MM	IA, BS, BL, JP, shadow	
Blue	Shadow, JP, IA	BL, BS, MM	15.08.21
Green	Shadow, BS, BL	IA, MM, JP	
Red	Shadow and all species		
Red-edge	Shadow and all species		
Near infrared	Shadow, JP, MM, BS	IA and BL	
Blue	Shadow, BS	BL, BS, MM	24.10.21
Green	Shadow	All species	
Red	Shadow, BS	JP, BL, MM, IA	
Red-edge	Shadow, JP IA	BS, BL, MM	
Near infrared	Shadow, JP, IA, BL	BS, BL, MM	

## Appendix C. Summary of Class Separability Using Mean Spectral Indices Features across the 3 Sampled Dates

**Bands**	**Separable Classes**	**Mixed Classes**	**Date**
Brightness	Shadow	All species	25.05.21
Maximum difference		All species, shadow	
NDVI	BS	JP, IA, BL, MM, shadow	
GCC	Shadow, MM, JP, BS	IA, BL	
RCC	BS	IA, MM, BL, JP, shadow	
Brightness	Shadow, JP, IA	BL, BS and MM	15.08.21
Maximum difference	All species and shadow		
NDVI	All species and shadow		
GCC	BL, BS, JP, MM	Shadow, IA	
RCC	All species and shadow		
Brightness	MM	BL, BS, IA, JP, shadow	24.10.21
Maximum difference	IA, BS, MM	shadow, BL, JP	
NDVI	BS, MM, JP, IA	BL, shadow	
GCC	BL, IA	JP, shadow/ BS, MM	
RCC	BS, MM, BL	shadow, JP, IA	

## Appendix D. Summary of Class Separability Using Mean Textural Features across the 3 Sampled Dates

**Bands**	**Separable Classes**	**Mixed Classes**	**Date**
Contrast	Shadow	All species	25.05.21
Correlation		JP, shadow, BS, MM/BL, IA	
Dissimilarity		All classes	
Entropy	BS	IA, BL, MM, JP, shadow	
Standard deviation	Shadow	All species	
Contrast	IA	BS, BL, MM, JP, shadow	15.08.21
Correlation		All classes	
Dissimilarity		All classes	
Entropy	Shadow, JP, BS	IA, MM, BL	
Standard deviation		JP, shadow/ MM, BS, BL, IA	
Contrast	Shadow	All species	24.10.21
Correlation	Shadow, JP	MM, IA, BS, BL	
Dissimilarity	Shadow	All species	
Entropy	Shadow, BS, BL, MM	JP, IA	
Standard deviation		All classes	
